# Integration and Implementation Challenges of State-Based Long-Term Services and Supports Financing Programs

**DOI:** 10.1093/geroni/igaa057.2531

**Published:** 2020-12-16

**Authors:** Eiileen Tell, Marc Cohen, Ben Veghte, Alexandra Bradley

**Affiliations:** 1 ET Consulting, LLC, Boston, Massachusetts, United States; 2 University of Massachusetts Boston, Boston, Massachusetts, United States; 3 Caring Across Generations, washington, District of Columbia, United States; 4 National Academy of Social Insurance, Washington, District of Columbia, United States

## Abstract

Several states are in the process of developing or implementing programs that use a social insurance approach to financing long-term services and supports (LTSS). The National Academy of Social Insurance developed a roadmap that highlights key design parameters for states to consider in crafting a program. It also outlines a range of vetted approaches states could adopt, and describes the building blocks and tradeoffs associated with a range of options. States that establish a new LTSS program will need to make decisions about the integration of the program with other payers and benefit programs as well as address concrete implementation challenges. This presentation summarizes these issues including coordination of benefits with other payers, Federal Medicaid funding issues, integration of LTSS and medical care, and challenges around program startup, administration and monitoring as well as evaluation and modification. Addressing such issues is critical to the success of these type of programs.

